# Genetic characterization of Spinocerebellar ataxia 1 in a South Indian cohort

**DOI:** 10.1186/s12881-014-0114-5

**Published:** 2014-10-25

**Authors:** Dhanya Kumaran, Krishnan Balagopal, Reginald George Alex Tharmaraj, Sanjith Aaron, Kuryan George, Jayaprakash Muliyil, Ajith Sivadasan, Sumita Danda, Mathew Alexander, Gaiti Hasan

**Affiliations:** National Centre for Biological Sciences, Tata Institute of Fundamental Research, Bangalore, Karnataka India; Manipal University, Manipal, 576104 India; Department of Neurological Sciences, Christian Medical College and Hospital, Vellore, Tamil Nadu India; Department of Community Health, Christian Medical College and Hospital, Vellore, Tamil Nadu India; Department of Clinical Genetics, Christian Medical College and Hospital, Vellore, Tamil Nadu India

**Keywords:** Spinocerebellar ataxia 1, Cohort, Pre-symptomatic, Founder mutation

## Abstract

**Background:**

Spinocerebellar ataxia type 1 (SCA1) is a late onset autosomal dominant cerebellar ataxia, caused by CAG triplet repeat expansion in the *ATXN1* gene. The frequency of SCA1 occurrence is more in Southern India than in other regions as observed from hospital-based studies. However there are no reports on variability of CAG repeat expansion, phenotype-genotype association and founder mutations in a homogenous population from India.

**Methods:**

Genomic DNA isolated from buccal mouthwash of the individuals in the cohort was used for PCR-based diagnosis of SCA1. Subsequently SNP’s found within the *ATXN1* loci were identified by Taqman allelic discrimination assays. Significance testing of the genotype-phenotype associations was calculated by Kruskal-Wallis ANOVA test with post-hoc Dunnett’s test and Pearson’s correlation coefficient.

**Results:**

By genetic analysis of an affected population in Southern India we identified 21 pre-symptomatic individuals including four that were well past the average age of disease onset of 44 years, 16 symptomatic and 63 normal individuals. All pre-symptomatic cases harbor “pure” expansions of greater than 40 CAGs. Genotyping to test for the presence of two previously identified SNPs showed a founder effect of the same repeat carrying allele as in the general Indian population. We show that SCA1 disease onset is significantly delayed when transmission of the disease is maternal.

**Conclusions:**

Our finding of early disease onset in individuals with a paternally inherited allele could serve as valuable information for clinicians towards early detection of SCA1 in patients with affected fathers. Identification of older pre-symptomatic individuals (n = 4) in our cohort among individuals with a shared genetic and environmental background, suggests that second site genetic or epigenetic modifiers might significantly affect SCA1 disease progression. Moreover, such undetected SCA1 cases could underscore the true prevalence of SCA1 in India.

## Background

Spinocerebellar ataxias (SCAs) are late onset, clinically and genetically heterogeneous neurodegenerative disorders broadly comprising of progressive cerebellar ataxia with variable involvement of the brain stem and spinal cord [1]. Amongst the SCAs, SCA1 was the first to be genetically characterized with an expansion of translated CAG trinucleotide repeats located within exon 8 of the *ATXN1* gene [2]. The repeat number is polymorphic and varies between 4 and 36 repeats. While in normal individuals, this repeat sequence may be occasionally interrupted by 1–3 CAT triplets, in SCA1 patients the repeats are expanded beyond 39 and are uninterrupted [3]. CAT interruptions among CAG repeats are postulated to prevent repeat expansion and contraction during DNA replication and repair. They are also known to enhance the stability of the repeat tract and thus render them non-pathogenic [4,5]. Therefore, molecular characterization of expanded repeats at the SCA1 locus is essential to allow both genetic counseling and to understand disease pathogenesis.

SCA1 occurs in diverse ethnic groups worldwide with varying prevalence [6–12]. In India, SCA1 accounts for 22% of ADCA (Autosomal Dominant Cerebellar Ataxia) [13]. Previous studies in the Indian population have studied the prevalence of SCA1 in hospital cases [13–17] where a higher frequency of SCA1 has been seen in South India [13,17]. One such study explored the genetic basis, such as founder mutations, frequency of large normal alleles (>30 repeats) and presence of CAT interruptions [13]. Small pockets of villages have been reported in Tamil Nadu in South India with a high prevalence of SCA1 [18]. But, genotypic studies, such as those done in the hospital cases, have not been conducted in these isolated communities of South India. Additionally, studies investigating disease onset in individuals living in homogenous conditions in order to understand phenotypic variability in individuals with shared genetic background and environment influences have not been conducted in India. We chose patients and their families from Adukkamparai, a village located in an area of South India, where a high prevalence of hereditary ataxia was recorded previously [18]. The study aimed to understand the genetic basis of ataxia in these patients and compare the manifestation of disease characteristics among individuals living in environmentally homogenous conditions.

## Methods

### Patient and families

All adults (48 males and 52 females) residing at Adukkamparai village in Vellore India, who agreed to participate (henceforth referred to as the cohort) were recruited in this study. The entire cohort, consisting of unaffected family members, members with progressive and non-progressive ataxia and first-degree relatives of the affected patients was evaluated. The clinical team comprised of investigators from departments of Community health, Neurology, Clinical genetics and Neurobiology from the two institutions. The Institutional review board of Christian Medical College and Hospital Vellore (Blue, Research and Ethics committee) approved the study (IRB Min no: 7692 dated 12.12.2011).

A detailed pedigree chart was constructed with emphasis on age of onset, severity, anticipation and paternal/maternal inheritance patterns using Haplopainter V1.043 software [19]. Other details such as ethnicity, geographic location of ancestors and occupation were also collected.

### DNA extraction

Buccal cells were collected from 100 individuals (48 males and 52 females) of the 15 families after informed consent. The cells were obtained from mouthwashes performed with 10 ml autoclaved double distilled water. They were stored at 4°C before processing for DNA extraction. DNA samples were obtained from cells that had been stored up to 20 days. Samples were centrifuged at 6000 rpm for 5 minutes so as to pellet the cells. DNA was isolated from the buccal epithelial cell pellet using the HiPurA Buccal DNA Miniprep Purification Spin columns (Hi-Media Pvt. Ltd, Mumbai, India) according to the manufacturer’s protocol.

### Analysis of CAG repeats and CAT interruptions

The isolated DNA was used to carry out PCR based assays for diagnosis of SCA1 with the rep1 and rep2 primers [20] and SCA2 specific primers (SCA2 A-B) [21] according to published protocols. Fragment analysis was performed with PCR products obtained by FAM-labeled rep2 primers and regular rep1 primers. The PCR products (1 μl) were mixed with 12 μl of formamide, and 0.7 μl LIZ-500 (Applied Biosystems, Life Technologies, Foster City, CA, USA) containing the molecular size standards for reference. Samples were denatured at 95°C for 10 min, placed on ice and subjected to capillary electrophoresis on an ABI genetic analyzer 3130xl. Data was analyzed using PeakScanner V2 software (Applied Biosystems, Life Technologies, Foster City, CA). The number of base pairs (bp) of CAG repeats was calculated by taking the size of the PCR product and subtracting 123 from it followed by dividing by 3; 123 bp is the size of the non-repeat region in the amplified product.

CAT interruption of the CAG repeats was assessed on the basis of restriction digestion of the PCR product with *Sfa*NI, which cleaves the PCR product in the presence of CAT trinucleotides [22].

### SNP analysis

Analysis of two SNPs, rs2075974 and rs1476464 in the *ATXN1* gene was performed by the Taqman allelic discrimination assay obtained from Applied Biosystems, Life technologies. Approximately 20–50 ng of genomic DNA was mixed with 12.5 μL of SNP genotyping master mix and 1.25 μl of 20X primer probe mix (Life technologies pre-designed SNP genotyping assay, part number C__16167072_10 for rs2075974 and part number C__7615001_10 for rs1476464) and run on the Applied Biosystems 7500 fast real time PCR system. The cycling temperatures were as follows: denaturation at 95°C for 10 minutes followed by 40 cycles of 95°C for 15 sec and 60°C for 1 minute. A particular SNP is identified by the corresponding amplification peak and is confirmed by the allelic calls generated by the 7500-software v2.0.5.

### Statistical analysis

The relationship between age of onset and inheritance with CAG repeats was calculated by Kruskal-Wallis ANOVA test with post-hoc Dunnett’s test. Pearson’s correlation coefficient was used to test linear association between repeat length and age of onset. Chi-square test and one-way ANOVA was used to test significance of qualitative data. Significance was calculated using the GraphPad Prism 6.0 software and results were considered significant at P = 0.05 level.

## Results

### Clinical and pedigree analysis of the cohort

**Figure 1:**
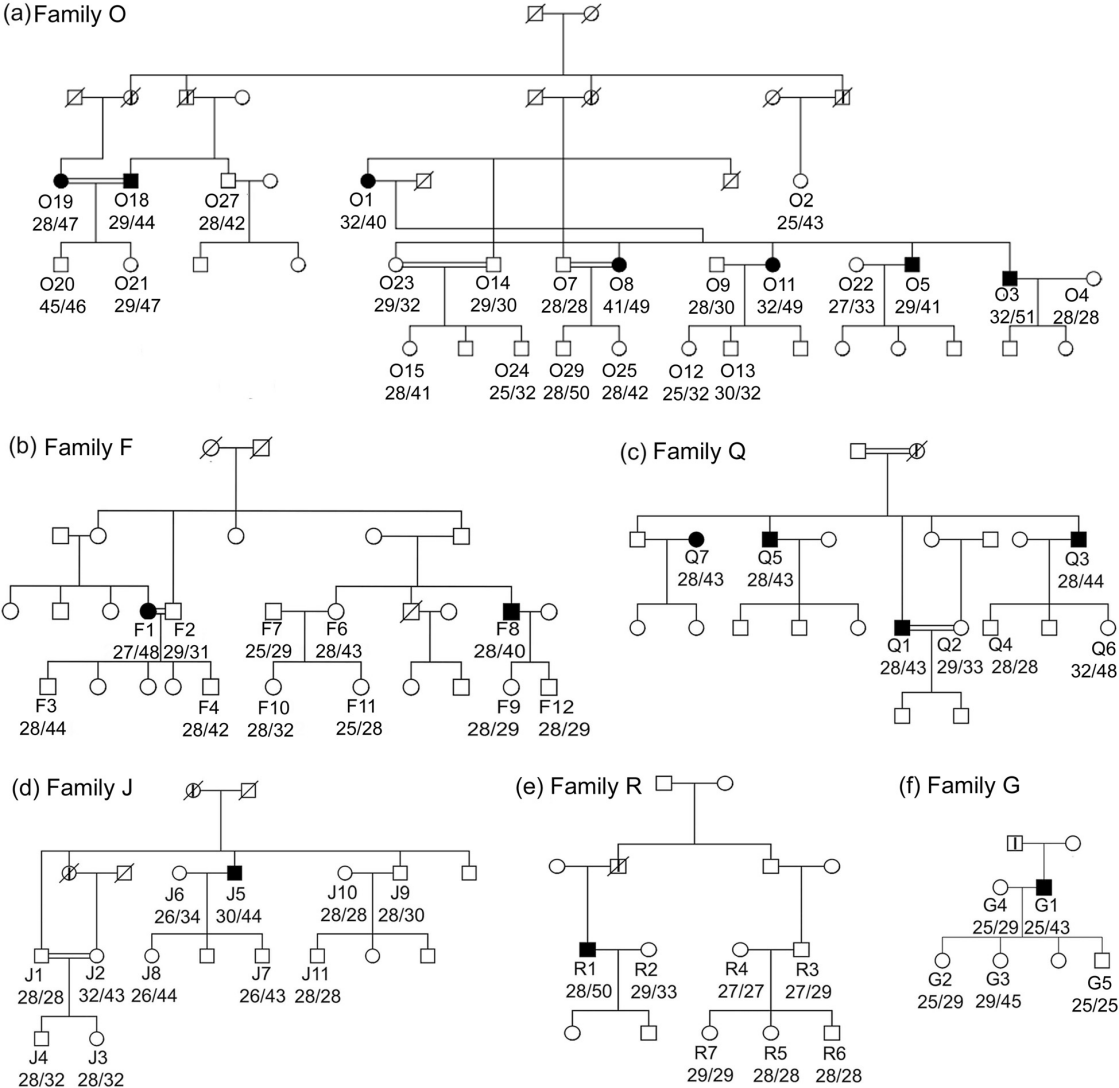
**Pedigree of six affected SCA1 families.** **a)** family O, **b)** family F, **c)** family Q, **d)** family J, **e)** family R and **f)** family G. Individuals (males as squares and females as circles) are represented as follows; *black-filled symbols:* those who were clinically affected; *symbols with a vertical line:* affected by hearsay; symbols with a line across: expired; *open symbols:* clinically unaffected. Texts below the symbols indicate the individual code followed by allele1/allele2 sizes.

All 100 individuals studied were residents of Addukkamparai for 20 years or more. These individuals belong to a group that have been practising consanguineous marriage for generations. Amongst this group we identified 16 individuals from 6 families with clinical symptoms of ataxia (Figure 1) after a comprehensive examination of cranial nerves, motor-sensory systems, extrapyramidal systems, peripheral nervous system and neurophthalmogic examination. Family O (Figure 1a) had the maximum number of clinically affected individuals (n = 7). Two families, F and Q, had 2 and 4 affected individuals respectively (Figure 1b and c) and 3 families, J, R and G had a single affected individual (Figure 1d, e and f).

### Genetic analysis of the cohort

**Figure 2:**
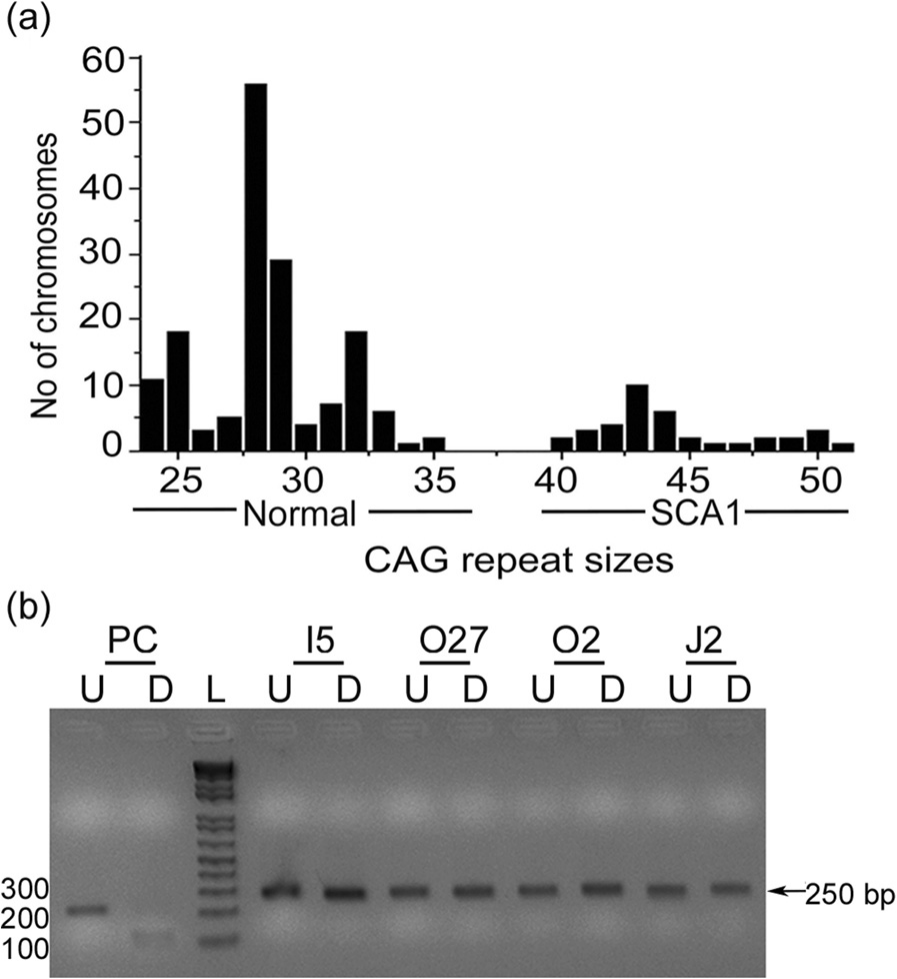
**CAG repeats distribution and analysis of CAT interruptions in ATXN1 gene. (a)** Distribution of CAG repeats in normal and expanded SCA1 alleles at the ATXN1 locus. **(b)** PCR products of *ATXN1* gene before (U; undigested) and after (D, digested) digestion with *SfaN1* in the pre-symptomatic individuals I5, O2, O27 and J2. PC denotes positive control.

After PCR based analysis for detection of *ATXN1* and *ATXN2* CAG repeats the diagnosis of SCA1 in the families was confirmed. The number of SCA1 repeats ranged from 24 to 51 in the 100 individuals recruited in this study (Figure 2a). Repeat sizes of 39 or more, characteristic of SCA1, were seen in 37 individuals. Among these individuals, 16 (43.24%) had clinical symptoms of ataxia with the youngest symptomatic individual being 30 years old, but 21 (56.75%) did not show any symptoms. These were classified as pre-symptomatic. Among the pre-symptomatics, 4 individuals were older than 45 years at the time of evaluation, the oldest being 60 years old. The repeat sizes of older pre-symptomatic individuals were 25/43 (O2, Figure 1a), 28/42 (O27; Figure 1a), 32/43 (J2; Figure 1d) and 24/43 (I5; not shown). These patients including the oldest among them, a 60 year old female (O2), did not show any clinical features of the symptomatic individuals such as gait ataxia, temporalis and masseter wasting, bucco-oral and limb dystonia, night cramps, dysarthria, pale discs, stare and peripheral neuropathy. To understand the possible contribution of environmental factors to the absence of SCA1 symptoms in these individuals (n = 4), we compared their lifestyle with that of symptomatic individuals in the same age bracket of greater than 45 years (n = 13). The occupation of such pre-symptomatic and symptomatic individuals and their use of tobacco and alcohol were categorized (Table 1). All symptomatic females (n = 5) were housewives (non-smokers and no alcohol use) with occasional farming activity. Their lifestyle and occupation appeared identical to the older pre-symptomatic females (n = 3). The symptomatic males (n = 8) had a range of occupations such as farming (n = 1), shopkeeper, small business etc. (n = 7). Regular smoking and occasional alcohol use was observed in 4 symptomatic males. The pre-symptomatic older male (O27) had no history of smoking or alcohol use. Based on these data no obvious differences in the lifestyle or dietary habits of the older pre-symptomatic individuals and the symptomatic individuals could be ascertained.

**Table 1:** **Comparison of occupation and lifestyle habits of older pre-symptomatic individuals with symptomatic SCA1 patients of similar age**

**Occupation/lifestyle**	**Pre-symptomatic females**	**Symptomatic females**	**Pre-symptomatic males**	**Symptomatic males**
House-wife	3 (J2, I5, O2)	5 (O11, O8, O19, Q7, O1)	-	-
Farming and occasional contract laborers	3 (J2, I5, O2)	4 (O11, O8, O19, O1)	1 (O27)	1 (O18)
Other occupation	-	-	-	7 (O5, F8, Q3, Q1, J5, G1, Q5)
Tobacco use (smoking)	-	-	-	2 (O18, Q5)
Alcohol consumption (occasional)	-	-	-	2 (O5, F8)

DNA from all 21 pre-symptomatic patients was tested for CAT interruptions using *Sfa*NI. Restriction enzyme *Sfa*NI [recognition sequence: 5’-GCATC(N)_5_3’] cleaves a CAT interrupted CAG repeat [22]. We found that all pre-symptomatic individuals, including the four elderly pre-symptomatics, harbored “pure” CAG expansions (Figure 2b).

In conclusion, normal range of repeats (24 to 35) was seen in 63 individuals and expansions ranging from 41–51 were observed in 37 individuals (16 symptomatic and 21 pre-symptomatic). Homozygous states of repeat expansions were seen in 2 individuals with genotypes of 41/49 (O8) and 45/46 (O20). O20 (20 years old) is pre-symptomatic and O8 (43 year old) symptomatic for SCA1 (Figure 1a). O19 (43 years old) with repeat size 28/47 shows symptoms as severe as O8, indicating that homozygosity does not contribute to disease severity. It is previously known that the disease manifestation in homozygous condition is contributed by the size of the larger allele and the smaller allele makes no contribution [7,18].

The remaining 35 patients were heterozygous for expanded CAG repeats. Significant changes in repeat numbers either expansions or contractions - were not observed between generations except in family Q (Figure 1c), where Q6 with the genotype 32/48 has an expansion of 4 CAG’s from her father Q3 (28/44). Increase in size of CAG repeats when transmitted from affected fathers is known in SCA1 [22,23].

### Correlation of Genotype with the observed Phenotypes

**Figure 3:**
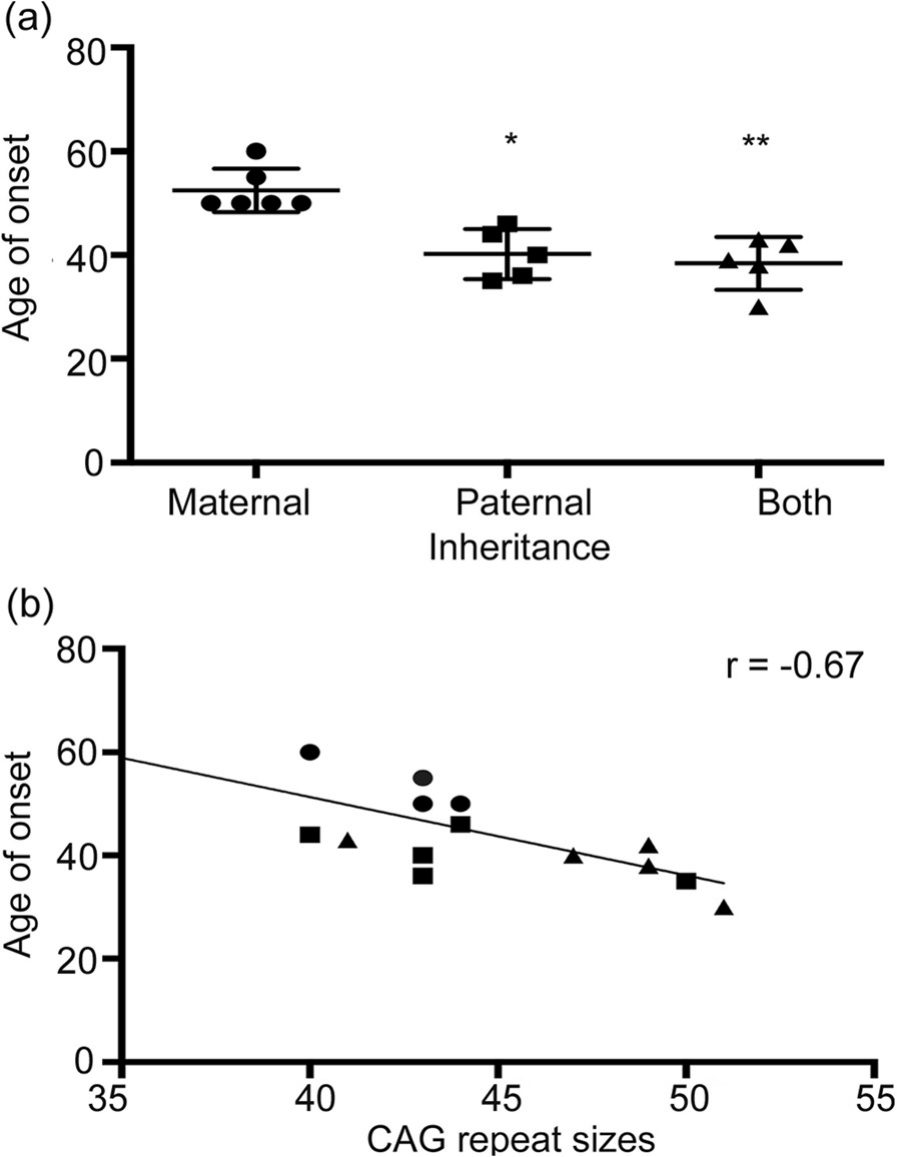
**Genotype-phenotype correlation among the SCA1 affected individuals. (a)** Relationship between inheritance pattern and age of onset of the disease. Data was analyzed using one-way ANOVA with Dunnett’s multiple comparison test (*P = 0.0015 and **P = 0.005). **(b)** Inverse correlation of age of onset with respect to the CAG expansions in the symptomatic patients (r^2^ = 0.45 and P = 0.002), where individuals with maternally inherited alleles are represented as circles, paternally inherited alleles as squares and inheritance from both parents as triangles.

Based on inheritance of the mutant gene we classified patients into three groups as follows: Group 1 (n = 6), with a maternally inherited mutated allele; Group 2 (n = 5), with a paternally inherited mutated allele and Group 3 (n = 5), in which both the parents were affected. The mean age of onset of disease in group 1 was 52.5 ± 4.18 years, whereas in group 2 it was 40.2 ± 4.81 years and in group 3 it was 38.6 ± 5.17 (Figure 3a). A significant difference was observed in the ages of onset between the groups 1, 2 and 3 by Kruskal-Wallis ANOVA test (P = 0.0004). As significant results were observed we compared group 1 with group 2 and 3 by ordinary ANOVA with post hoc Dunnett’s test. Group 1 was found to be significantly different from group 2 (P = 0.0015) and group 3 (P = 0.005), whereas no significant difference was observed between group 2 and 3 (P = 0.8). Thus, patients with maternally inherited alleles in the cohort show a later age of onset when compared to those with paternally inherited alleles. When both parents were affected, the age of onset was determined by the paternal allele. Similar difference in the age of onset between paternal and maternal inheritance of the CAG expanded chromosome has been observed in Huntington’s disease [24,25]. To the best of our knowledge a similar finding has not been described for SCA1 to date.

Correlation of CAG repeats size with age of onset and inheritance (Figure 3b) showed that both maternal and paternally transmitted alleles harbored repeat sizes in the range of 40–45 (shown as circles for maternally transmitted and squares for paternally transmitted alleles in Figure 3b). Individuals with both parents affected (triangles in Figure 3b) had longer repeats. Similar to previous studies [26] the age of onset of ataxia in the symptomatic individuals (n = 16) correlated negatively with greater repeat numbers (r = −0.67, P = 0.002), indicating that 45% (R^2^ = 0.45) of the variation in age of onset is accounted by the size of the repeat (Figure 3b).

### Founder mutation

**Figure 4:**
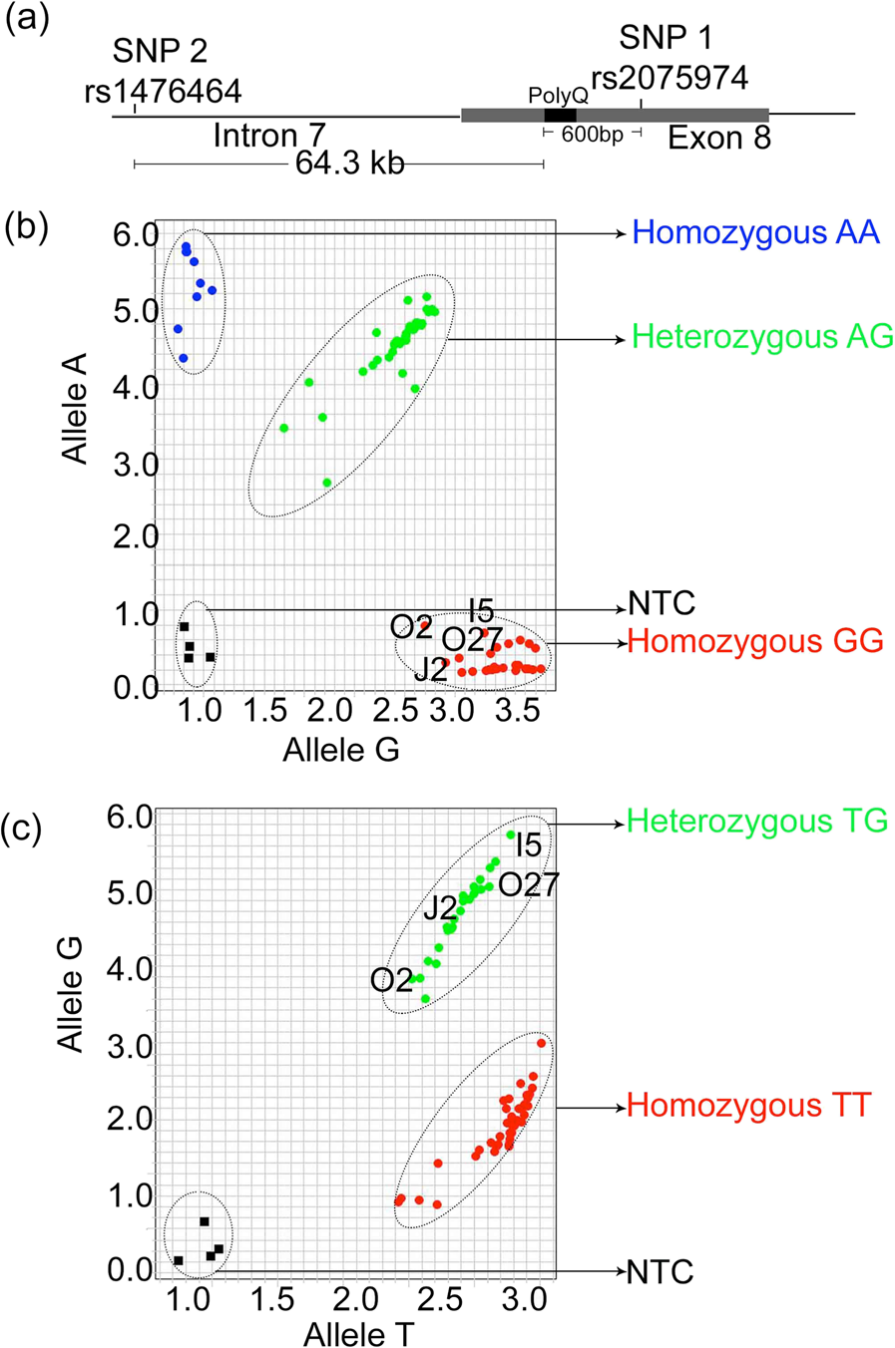
**Genotyping of SCA1. (a)** Schematic diagram showing the position of the two SNPs within the *ATXN1* gene. **(b)** Representative allelic discrimination plots for the rs2075974 (SNP1) showing homozygous for A in blue and homozygous for G in red and heterozygous (AG) in green and negative control (NTC) in black for the samples from the cohort. **(c)** Representative allelic discrimination plots for the rs1476464 (SNP2) showing the allele calls for homozygous for T in red and heterozygous (TG) in green, and the negative controls in black for the samples from the cohort.

To understand if there was a founder effect of the mutation in this population we looked at the two SNP’s (represented in Figure 4a) surrounding the CAG expansions. Allelic discrimination plots for the SNP’s are shown in Figure 4b, c. SNP1 (rs2075974) and SNP2 (rs1476464) for 35 patients, including the pre-symptomatic elder individuals O2, O27, J2 and I5 mentioned above, showed a clear association to the G allele. The association of the two SNPs with normal and SCA1 patients were significantly different (P = 0.0001; Table 2). The allele G showed association with the expanded chromosome at SNP1 and SNP2 (Table 2). Presence of the G allele at both SNP1 and 2 in a patient with homozygous mutant alleles (O20) further supports our conclusion that the alleles G-G are linked to the disease locus in this cohort, as previously established for other Indian SCA1 families [13].

**Table 2:** **Association of the SNPs between normal and SCA1 individuals of Adukkamparai**

**SNP**		**Normal (frequency)**	**SCA1 (frequency)**	
			**Pre-symptomatic**	**Symptomatic**
**SNP1**	AA	12/59 (0.20)	0/17 (0)	0/16 (0)
	GG	12/59 (0.20)	11/17 (0.64)	8/16 (0.5)
	AG	35/59 (0.59)	6/17 (0.35)	8/16 (0.5)
**SNP2**	GG	0/59 (0)	1/17 (0.05)	0/16 (0)
	TT	59/59 (1)	0/17 (0)	0/16 (0)
	TG	0/59 (0)	16/17 (0.94)	16/16 (1)

## Discussion

In this study, we identify both pre-symptomatic and symptomatic SCA1 individuals in Adukkamparai using a non-invasive screening strategy. Our study shows important phenotype-genotype correlations in a population practicing consanguineous marriage for generations. The range of expanded alleles (40–51) in patients from this village was comparable to those that have already been reported in the Indian population [14,16–18]. As compared with other studies, where 66% of the variation in age of onset is accounted by CAG repeat size (26), our data indicate that the age of onset in Adukkamparai patients is only accounted to 45% by the repeat size. Moreover, the age of onset in individuals with paternally inherited alleles is earlier than in individuals with maternally inherited alleles and this appears to be independent of the size of CAG repeats. No marked anticipation was observed.

The prevalence of SCA1 varies in different regions and has been attributed to the presence of a higher frequency of large normal alleles (>30 repeats) and due to single interruptions in normal alleles within the population [12,13,22,27]. Trinucleotide disorders are known to harbor alleles of large size in the normal range (large normal alleles) that are unstable and therefore prone to expansions into the disease range. Thus a higher frequency of large normal alleles in a population is an indirect measure of the prevalence of the disease [27–29]. In our study we did not observe a high frequency of large normal alleles (0.21) as compared to the frequency of small normal alleles (0.78). African [6] and Siberian Sakha [7] cohorts having a high prevalence of SCA1 have also reported a low frequency of large normal alleles. The Siberian Sakha population, however had a higher proportion of individuals with single CAT interruptions within the normal alleles [12]. It is hypothesized that loss of CAT interruptions predisposes a stable allele to further expansions [22]. We therefore assume that the high prevalence of SCA1 we observe at Adukkamparai could be contributed by expansions of non-affected alleles carrying single CAT interruptions, which were lost over time.

When the chromosomal background of the expanded *ATXN1* was tested for SNPs known to be associated with CAG expansions, the results correlated with those from previous studies carried out in SCA1 patients from Southern and Northern India [13]. An association of the genotypes G and G at loci (rs1476464 and rs2075974) was found in these patients. These G-G alleles can thus provide additional markers for the diagnosis of SCA1. This is the first study highlighting this association of two SNP’s (G-G) within an ethnic community in Tamil Nadu. Therefore, although prevalence of SCA1 in southern India seems to be more, there does not seem to be a different founder from North India.

Very recent implementation of personalized gene silencing by SNP-directed approaches is being considered as a potential therapeutic strategy in Huntington’s disease, another triplet repeat disorder [30]. SNP-directed gene silencing approaches are likely to be tried in future in the context of personalized medicine. By identifying SNPs associated with SCA1 in the cohort this study paves the way for selective silencing of disease alleles using SNP-targeted approaches in the future.

The existence of four individuals in the cohort who are asymptomatic and beyond the threshold age of onset could be attributed to differences in environmental conditions. However, this seems unlikely given the apparent uniformity of life style and dietary habits of both symptomatic and asymptomatic individuals within a small geographic region. Three asymptomatic individuals are females who share similar lifestyles (house-wives) and occupation (farming) with symptomatic females from the cohort. The male asymptomatic individual (age 50 years) is a farmer and an occasional contract laborer, and shares his lifestyle and occupation with a symptomatic male. The absence of smoking and alcohol usage among all the pre-symptomatics might suggest an apparent “protective” life style. However, the numbers are currently too few for a statistical comparison between the two groups. Rather, these asymptomatic individuals suggest the presence of genetic modifiers such as SNPs and epigenetic changes for the pathogenesis of SCA1 and in manifestation of symptoms. Previous studies in other populations have reported similar presymptomatic individuals beyond the age of onset and have suggested incomplete penetrance of the mutation as the cause [7].

## Conclusions

Genome wide linkage scans [31,32], exome sequencing [33] and targeted gene sequencing [34] by Next Generation Sequencing could be employed for identifying the genetic modifiers proposed from the findings of this study and previous studies. Further molecular and genetic studies in individuals from the Adukkamparai cohort and the other cohorts with greater numbers of pre-symptomatic individuals carried out over time will aid in providing a mechanistic perspective and natural history of disease progression. These studies would eventually help in determining and targeting therapeutic interventions based on various stages of the disease.
